# Nonlinear Digital Self-Interference Cancellation in In-Band Full-Duplex Systems with Complex-Valued Temporal Convolution

**DOI:** 10.3390/s26144532

**Published:** 2026-07-17

**Authors:** Jun Chen, Xiaobo Wang, Rui Wang, Hongzhi Zhao

**Affiliations:** 1 Southwest China Institute of Electronics Technology, Chengdu 611731, China; johnus@126.com; 2 National Key Laboratory of Wireless Communications, University of Electronic Science and Technology of China, Chengdu 611731, China; 202212220624@std.uestc.edu.cn (X.W.); 202521220416@std.uestc.edu.cn (R.W.)

**Keywords:** in-band full-duplex systems, nonlinear self-interference, self-interference cancellation, complex-valued representation, temporal convolutions, integrated sensing and communication

## Abstract

In-band full-duplex (FD) technology has emerged as a promising solution to the growing demand for higher spectrum efficiency. By enabling simultaneous transmission and reception at the same frequency, FD-based integrated sensing and communication (ISAC) architectures offer potential advantages in capacity, latency, and spectral efficiency. However, non-ideal hardware in transceivers introduces nonlinear self-interference (SI), which limits the practical deployment of FD systems. Conventional polynomial models are widely used to characterize nonlinear SI, but their computational complexity grows quadratically with the nonlinear order. To address these challenges, a complex-valued temporal convolutional network (CV-TCN) with wavelet activation function is proposed for nonlinear SI modeling in this paper. The CV-TCN canceller achieves stronger nonlinear SI cancellation (SIC) performance with lower inference complexity. Simulation results demonstrate that the proposed model surpasses traditional polynomial-based and other cancellers in nonlinear SIC performance with higher parameter efficiency. Moreover, the CV-TCN canceller suppresses the residual SI power by around 7.95 dB after linear SIC, leaving the residual SI 2.23 dB above the noise floor.

## 1. Introduction

Full-duplex (FD) communication enables simultaneous transmission and reception within the same frequency band, making it a key candidate for improving spectral efficiency in integrated sensing and communication (ISAC) systems [1,2,3,4]. FD-ISAC, which integrates FD with ISAC, surpasses conventional ISAC architectures by enhancing communication capacity, eliminating sensing blind areas, and enabling seamless communication–sensing coordination [5]. However, such operation introduces strong self-interference (SI) which occurs when the transmitted signal leaks into the receiver [6]. Thus, mitigation of SI at the receiver is acknowledged as a primary challenge for the practical deployment of FD-based ISAC systems.

Nonlinear SI arises in FD systems when the SI signal propagates through non-ideal hardware components [7], including digital-to-analog converters (DACs), analog-to-digital converters (ADCs), low-noise amplifiers (LNAs) and power amplifiers (PAs). Typically, polynomial models are used to characterize nonlinear SI signals [7]. However, polynomial models suffer from substantial computational complexity and a large number of parameters, both of which grow quadratically with the nonlinear order. For this reason, various neural networks (NNs) have been proposed to model nonlinear SI signals [8,9].

Existing NNs for nonlinear SI cancellation (SIC) mainly fall into two categories which are real-valued NNs (RVNNs) and complex-valued NNs (CVNNs) [10]. CVNNs are well-suited for signal processing in FD systems because they natively preserve amplitude and phase information for the baseband signal, offering better parameter efficiency and modeling accuracy than RVNNs [11]. First introduced in [12], NNs adopt complex-valued parameters for information processing. Subsequently, a complex-valued feed-forward NN (CV-FFNN) is introduced in [13] to model the nonlinear SI signal. The ladder-wise grid structure (LWGS) and the moving-window grid structure (MWGS) are proposed to reduce computational complexity of nonlinear SI modeling in [14]. The dual-neuron hidden layer NN (DN-HLNN) is proposed based on the CVNN structure in [15], which attains performance enhancement in terms of parameters and floating-point operations (FLOPs) over the existing CVNN-based cancellers.

To design CVNN-based cancellers with superior SIC performance and reduced inference complexity, a complex-valued temporal convolutional network (CV-TCN) with wavelet activation is proposed in this work. The proposed model uses causal temporal convolutions for sequence modeling, complex-valued operations for baseband signal processing, and Morlet wavelet activation for nonlinear SI fitting. Experiments show that CV-TCN outperforms existing methods in parameter efficiency and inference complexity. The main contributions of this work are summarized as follows.

- Firstly, a complex-valued temporal causal convolution NN is proposed, referred to as CV-TCN. The complex-valued parameter design reduces the number of model parameters, while temporal causal convolution lowers inference complexity.
- Secondly, a Morlet wavelet activation function is introduced to replace generic activation functions, which possesses time–frequency localization and multi-scale analysis capabilities, enhancing the network’s ability to fit nonlinear distortions.
- Finally, the performance of the proposed CV-TCN canceller is analyzed in three dimensions: nonlinear SIC performance, parameter efficiency, and inference complexity. Compared with existing NN-based models, CV-TCN achieves superior performance under the same parameter count or inference complexity.

The remainder of this paper is organized as follows. Section 2 describes the system model for FD systems. Related works and proposed CV-TCN are discussed in Section 3. Numerical simulation results are provided in Section 4, and Section 5 concludes this paper.

## 2. System Model

This section presents the signal model for an FD transceiver, including nonlinear distortion introduced by non-ideal hardware components. Then, the frameworks for linear and nonlinear SIC are presented.

### 2.1. Full-Duplex Systems

**Figure 1:**
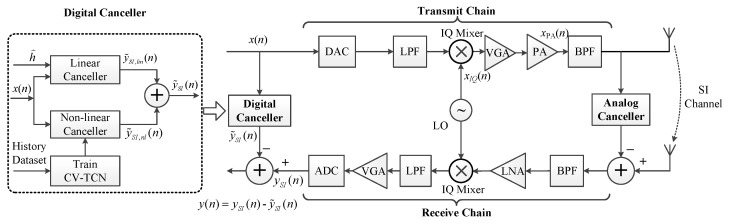
Full-duplex transceiver system model.

The FD transceiver system diagram with digital SIC is depicted in Figure 1. FD technology enables simultaneous signal transmission and reception over the same frequency with the help of SI cancellers.

At time instant *n*, the transmitted complex baseband signal x(n) passes through a set of analog components. Specifically, the digital signal is first converted to an analog waveform via a DAC, then frequency up-conversion is conducted through an in-phase and quadrature (IQ) mixer. With an ideal DAC, the equivalent baseband expression for the output signal of the IQ mixer is given by [8]

(1) $$x_{IQ}\left(n\right)=\frac{1}{2}\left(1+\alpha e^{j\phi }\right)x\left(n\right)+\frac{1}{2}\left(1-\alpha e^{j\phi }\right)x*\left(n\right),$$

where α and ϕ denote the gain and phase imbalance factors of the IQ mixer, respectively. The amplitude of $x_{IQ}$ is adjusted by a variable gain amplifier (VGA), and then the signal is amplified by the PA, which will introduce nonlinearity to the transmitted signal. In practice, the parallel Hammerstein model is commonly employed to characterize the nonlinear distortions, which is given by [8]

(2) $$x_{PA}\;\left(n\right)\;=\;\sum_{\underset{p\;odd}{p=1,}}^{P}\;\sum_{m=0}^{M}h_{m,p}x_{IQ}{\left(n-m\right)}^{\frac{p+1}{2}}\;x_{IQ}^{*}{\left(n-m\right)}^{\frac{p-1}{2}},$$

where $h_{m,p}\in C$ is the impulse response of the parallel Hammerstein model, *M* represents the memory depth, and *P* is the nonlinear order of the PA. Assuming the low-pass filter (LPF), band-pass filter (BPF), and analog-to-digital converter (ADC) are ideal, the down-converted SI signal at the receiver can be expressed as [8]

(3) $$y_{SI}\left(n\right)=\sum_{l=0}^{L_{SI}-1}h_{SI}\left(l\right)x_{PA}\left(n-l\right),$$

where $h_{SI}\left(l\right)$ is the impulse response of the SI channel, and $L_{SI}$ represents the memory length of the SI channel. By substituting Equation (2) into Equation (3), $y_{SI}\left(n\right)$ is rewritten as [15]

(4) $$y_{SI}\;\left(n\right)\;=\;\sum_{\underset{p\;odd}{p=1,}}^{P}\;\sum_{q=0}^{p}\;\sum_{t=0}^{T-1}\;h_{p,q}\;\left(t\right)x{\left(n-t\right)}^{q}x*\;{\left(n-t\right)}^{p-q}\;,$$

where $T=M+L_{SI}$ is the total memory length of the combined system, and $h_{p,q}\left(m\right)\in C$ denotes the system impulse response for the integrated effects of PA, IQ mixer, and SI channel.

### 2.2. Linear and Nonlinear Cancellations

For digital SIC, the SI waveform is decomposed into linear and nonlinear components, which is written as

(5) $${\tilde{y}}_{SI}\left(n\right)={\tilde{y}}_{SI,lin}\left(n\right)+{\tilde{y}}_{SI,nl}\left(n\right),$$

where ${\tilde{y}}_{SI,lin}\left(n\right)$ and ${\tilde{y}}_{SI,nl}\left(n\right)$ denote the linear and nonlinear components of SI, respectively.

The linear component ${\tilde{y}}_{SI,lin}\left(n\right)$ can be obtained by setting P=1 and q=1 in Equation (4), which is a linear time-domain regression model [15]

(6) $${\tilde{y}}_{SI,lin}\left(n\right)=\sum_{t=0}^{T-1}h_{1,1}\left(t\right)x\left(n-t\right),$$

where the inference FLOPs and parameters of ${\tilde{y}}_{SI,lin}$ are 10T−2 and 2T [9], respectively.

The nonlinear component ${\tilde{y}}_{SI,nl}$ is formulated as a nonlinear function of historical baseband signal x(n), which is expressed as

(7) $${\tilde{y}}_{SI,nl}\left(n\right)=F\left\{x\left(n\right),x\left(n-1\right)\ldots ,x\left(n-T+1\right)\right\},$$

where F{·} denotes the nonlinear function learned by the cancellation model.

After digital SIC, the residual SI is given by

(8) $$y\left(n\right)=y_{SI}\left(n\right)-{\tilde{y}}_{SI}\left(n\right).$$

To quantitatively evaluate the SIC performance, the digital SIC ratio in dB is defined as [15]

(9) $$C_{SIC}=10\log_{10}\left(\frac{\sum_{n}{\left|y_{SI}\left(n\right)\right|}^{2}}{\sum_{n}{\left|y(n)\right|}^{2}}\right)\;.$$

### 2.3. Polynomial-Based Canceller

The polynomial-based nonlinear SIC aims to estimate the equivalent channel coefficients $h_{p,q}\left(m\right)$ in Equation (4). The total number of parameters of the polynomial model is written as [9]

(10) $$P_{P}=2T\left(\frac{P+1}{2}\right)\left(\frac{P+1}{2}+1\right)-2T,$$

which increases quadratically with the PA nonlinearity order. The total inference FLOPs of Equation (4) can be expressed as [9]

(11) $$\begin{array}{cc} F_{P,Mul} & =3T\left\{\left(\frac{P+1}{2}\right)\left(\frac{P+1}{2}+1\right)-1\right\},\\ F_{P,Add} & =7T\left\{\left(\frac{P+1}{2}\right)\left(\frac{P+1}{2}+1\right)-1\right\},\\ F_{P} & =F_{P,Mul}+F_{P,Add}, \end{array}$$

where $F_{P,Mul}$ and $F_{P,Add}$ represent the FLOPs of multiplication and addition operations, respectively. $F_{P}$ is the total FLOPs of the polynomial-based canceller.

## 3. Proposed Methods

In this section, the proposed CV-TCN architecture is first introduced. The computational complexity of the CV-TCN for inference is then analyzed.

### 3.1. The CV-TCN Model

**Figure 2:**
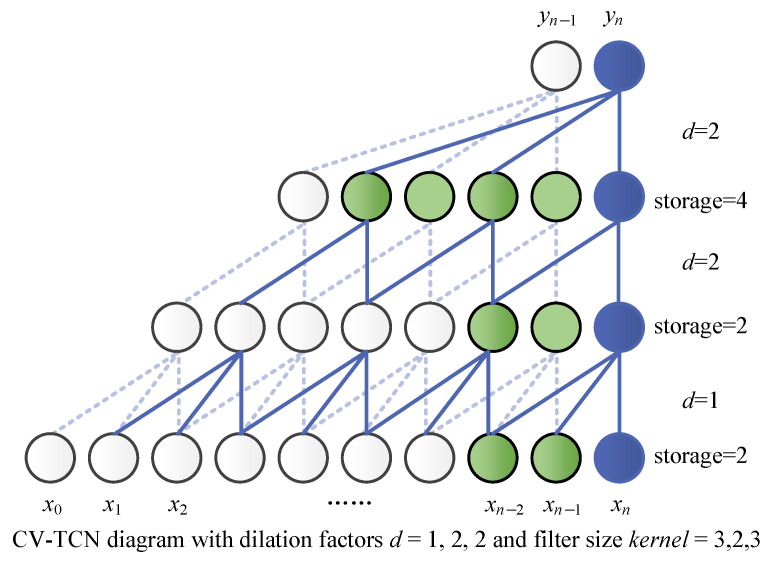
CV-TCN model’s structure. Only the blue nodes need to be computed to predict the SI signal when $x_{n}$ comes and the trade-off is additional data storage for green nodes during inference for CV-TCN.

As presented in Figure 2, input data is formatted into a complex-valued 1D sequence, and then processed by a 1D convolutional layer, followed by an activation function. Dilation is employed to enlarge the receptive field of the model while keeping the parameters and FLOPs the same. Finally, at the output layer of the CV-TCN, the I/Q components of the nonlinear SI signal are estimated by a complex-valued output.

#### 3.1.1. Complex-Valued Representation

In digital communications, baseband signals are typically represented as complex-valued. Thus, it is favorable to process these signals effectively and accurately within the complex domain, capturing amplitude and phase information simultaneously. Inspired by this, CVNNs are incorporated into the CV-TCN framework.

As illustrated in Figure 2, the CV-TCN model employs 1D convolutional layers with complex-valued parameters and Morlet activation functions. The utilization of complex-valued weights not only reduces the number of parameters but also enhances data representation capabilities.

#### 3.1.2. Temporal Causal Convolution

Temporal causal convolutions in [16] are utilized in the 1D convolutional layers to decrease the inference complexity. For example, only the blue nodes need to be computed to predict the SI signal when $x_{n}$ comes in Figure 2. However, the trade-off is additional data storage for green nodes during inference. For the *i*-th convolutional layer, the additional storage size $M_{CV\text{-}TCN}^{i}$ can be written as

(12) $$M_{CV\text{-}TCN}^{i}=2C_{in}^{i}\times \left(K^{i}-1\right)\times d^{i},$$

where $C_{in}^{i}$, $K^{i}$, and $d^{i}$ represent the input channel number, kernel size, and dilation size of the *i*-th convolutional layer, respectively.

#### 3.1.3. Morlet Activation Function

The Morlet is employed as the activation function for CV-TCN, stemming from its strengths in signal analysis, including good localization and multi-scale analysis properties. The Morlet wavelet is a smooth, continuous, symmetric, bounded, and non-monotonic activation function [17,18].

Defined as a sine wave modulated by a Gaussian function, the Morlet wavelet is mathematically written as

(13) $$W\left(x\right)=\sin\left(x\right)\cdot \exp\left(-\frac{x^{2}}{4\gamma^{2}}\right),\;$$

and if we let $a=-1/(4\gamma^{2})$, then Equation (13) can be rewritten as

(14) $$W\left(x\right)=\sin\left(x\right)\cdot \exp\left(ax^{2}\right).\;$$

The Morlet function is zero-centered and odd-symmetric about the origin. This zero-centered characteristic aligns well with the zero-mean nature of the SI signal, enabling the CV-TCN to model the nonlinear SI effectively. Assuming that each real operation (e.g., multiplication, division, addition, subtraction, sine, logarithm, and exponentiation) costs one FLOP [19], five FLOPs are required to compute the Morlet wavelet activation function. Since the Morlet function W(x) is applied element-wise to the real and imaginary parts, the output for a complex-valued input $x_{1}=a+jb$ is given by

(15) $$W\left(x_{1}\right)=W\left(a\right)+jW\left(b\right).$$

### 3.2. Computational Complexity for CV-TCN

The FLOPs required by the CV-TCN model are denoted as $F\;=\;\sum_{i}F_{CV\text{-}TCN}^{i}$, where $F_{CV\text{-}TCN}^{i}$ represents the inference FLOPs for the *i*-th 1D temporal convolutional layer. Accounting for both the complex-valued convolution and the activation function, $F_{CV\text{-}TCN}^{i}$ is formulated as

(16) $$\begin{array}{cc} Mul^{i} & =C_{out}^{i}\times C_{in}^{i}\times K^{i},\\ Add^{i} & =C_{out}^{i}\times C_{in}^{i}\times K^{i},\\ F_{CV\text{-}TCN}^{i} & =8Mul^{i}+2Add^{i}+2C_{out}^{i}F_{Act}^{i}\\ & =10C_{out}^{i}C_{in}^{i}K^{i}+2C_{out}^{i}F_{Act}^{i}, \end{array}$$

where $Mul^{i}$, $Add^{i}$, $C_{in}^{i}$, $C_{out}^{i}$, and $K^{i}$ denote complex-valued multiplication numbers, complex-valued addition numbers, input channel numbers, output channel numbers, and filter length of the *i*-th 1D temporal convolutional layer, respectively. $F_{Act}^{i}$ is the FLOPs of the activation function.

The total parameter numbers of the proposed CV-TCN is given by

(17) $$P\;=\;\sum_{i}P_{CV\text{-}TCN}^{i},$$

where $P_{CV\text{-}TCN}^{i}$ denotes the number of parameters for the *i*-th 1D temporal convolutional layer, and the value can be calculated by

(18) $$P_{CV\text{-}TCN}^{i}=2C_{out}^{i}\times \left(C_{in}^{i}K^{i}+1\right).$$

## 4. Experiments

In this section, the dataset and experimental setup are first described, followed by ablation studies on the Morlet activation function. Subsequently, we analyze the performance of the standard polynomial-based cancellers, existing NN-based cancellers, and the proposed CV-TCN canceller.

### 4.1. Datasets

Two public datasets employed in [8] (Dataset 1: https://github.com/abalatsoukas/fdnn (accessed on 1 March 2026)) and [20] (Dataset 2: https://github.com/abalatsoukas/CSI-full-duplex (accessed on 1 March 2026)) are utilized in this work. The datasets are measured data from a hardware FD testbed, which uses orthogonal frequency division multiplexing (OFDM) signal, quadrature phase shift keying (QPSK) modulation and passband bandwidths of 10 MHz and 20 MHz, respectively. To evaluate the CV-TCN’s performance with different bandwidths, modulations, carrier numbers and SI channels, one dataset is added which is generated via simulation. In this dataset, the PA is modeled with nonlinear order P=5 and memory depth M=3, and the propagation follows a 10-tap Rician channel.

The three datasets consist of 20,480 samples, of which 90% are reserved for training and the other 10% for testing. A summary of the system parameters [8,20,21] used to generate the datasets is provided in Table 1.

### 4.2. Experimental Setup for Dataset 1

The experiments for Dataset 1 are conducted with tap number T=13. This selection is based on the prior results that further increasing tap number yields minimal improvement in nonlinear SIC performance [8]. Consequently, the receptive field of the CV-TCN is configured to match this tap number. Similar to other NN-based cancellers, the CV-TCN processes the residual nonlinear SI signal after linear cancellation.

The proposed CV-TCN is implemented in Python 3.10 using the TensorFlow 2.10.0 and Keras 2.10.0. The model is optimized using the Adam optimizer [22] with a learning rate of 0.001, a batch size of 512, and 1000 training epochs. Jitter augmentation is adopted to enrich the training dataset, with a standard deviation of σ=0.1 and an augmentation factor of 2. The kernel weights of CV-TCN are initialized via the Glorot_uniform scheme, and all biases are initialized to zero. All CV-TCN experiments are trained with 10 independent random initializations for a fair comparison with baseline methods.

### 4.3. Activation Functions for Dataset 1

To evaluate the effect of activation functions, seven configurations are selected for the CV-TCN with receptive field T=13. As shown in Table 2, these configurations span a range from 78 parameters/314 FLOPs (tiny) to 1598 parameters/6460 FLOPs (huge), establishing a comprehensive testing scenario for activation function comparison.

Various activation functions are employed for ablation studies in the CV-TCN model, including ReLU, LeakyReLU, Mish, Tanh, Sigmoid, and Morlet wavelet with γ=π. All experiments are trained under identical optimization conditions to ensure that any observed performance differences are only caused by the choice of activation function. Table 2 also summarizes the performance and computational complexity of the CV-TCN cancellers across these configurations.

The Morlet wavelet achieves consistent performance gains across all model sizes, outperforming ReLU by 5.2–26.3% and Mish by 1.3–14.4%. The Morlet-ReLU differential peaks at 1.44 dB in the mini architecture, gradually decreasing to 0.39 dB in the large-scale models.

### 4.4. Comparison with Existing Models for Dataset 1

As shown in Table 3, the digital linear cancellation attains an approximate SIC ratio of 37.86 dB, while existing NN-based nonlinear cancellers can further reduce the SI power by around 6.1 dB to 8.0 dB. Particularly, the CV-TCN small canceller matches the performance of the RNN(16-16-16) [13] while decreasing the parameters from 1420 to 136 and FLOPs from 3106 to 670. It is important to note that the CV-TCN large canceller achieves a 7.95 dB nonlinear SIC ratio, which outperforms the KD-MCNN teacher model by 0.25 dB. Concurrently, the CV-TCN mini canceller surpasses DN-2HLNN(2-6) by 0.54 dB with a reduction of 10 parameters and 18 FLOPs.

**Figure 3:**
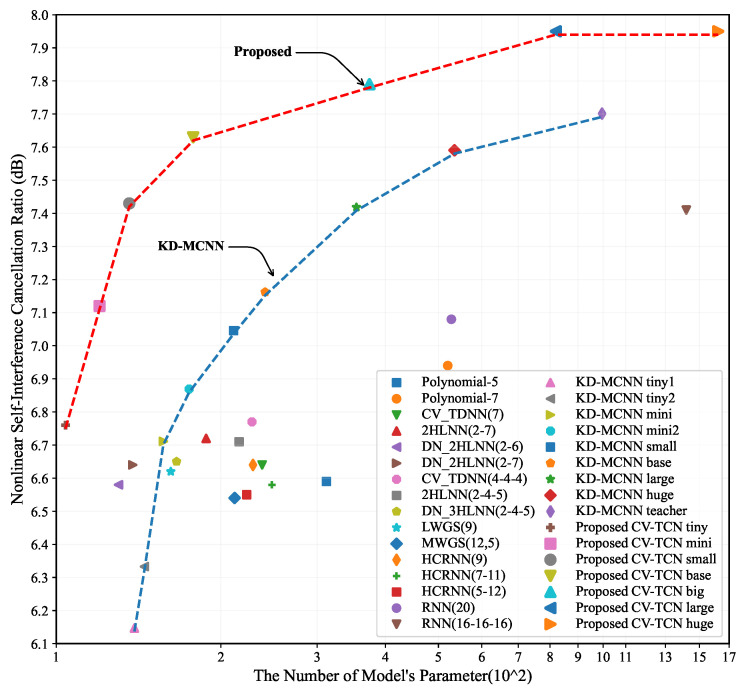
Nonlinear SIC ratio for proposed and existing models versus models’ parameters [9,15,19].

**Figure 4:**
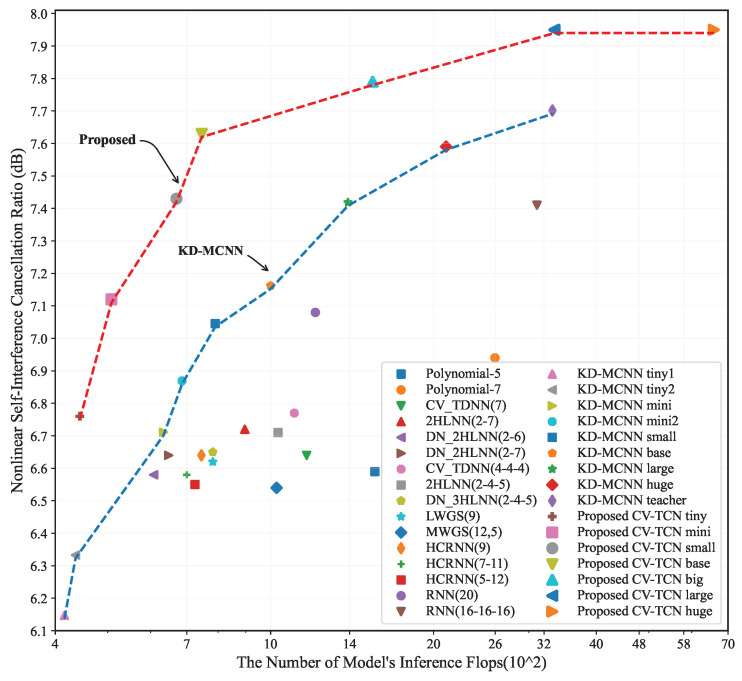
Nonlinear SIC ratio for proposed and existing models versus models’ inference FLOPs [9,15,19].

The relationship between the model’s parameters and nonlinear SIC is shown in Figure 3, while the relationship between inference FLOPs and nonlinear SIC is presented in Figure 4. These figures illustrate the effectiveness and efficiency of the proposed CV-TCN from the perspectives of nonlinear SIC performance, parameter reduction, and inference complexity reduction.

As depicted in Figure 3, the CV-TCN small canceller achieves a nonlinear SIC ratio of 7.43 dB with only 136 parameters, whereas the KD-MCNN large canceller requires 354 parameters to attain the same level of performance.

Furthermore, as shown in Figure 4, the CV-TCN cancellers exhibit superior performance compared with other NN-based cancellers under identical FLOP budgets. With only 56% inference FLOPs of the KD-MCNN teacher canceller, the CV-TCN big canceller achieves the same level of nonlinear SIC performance.

**Figure 5:**
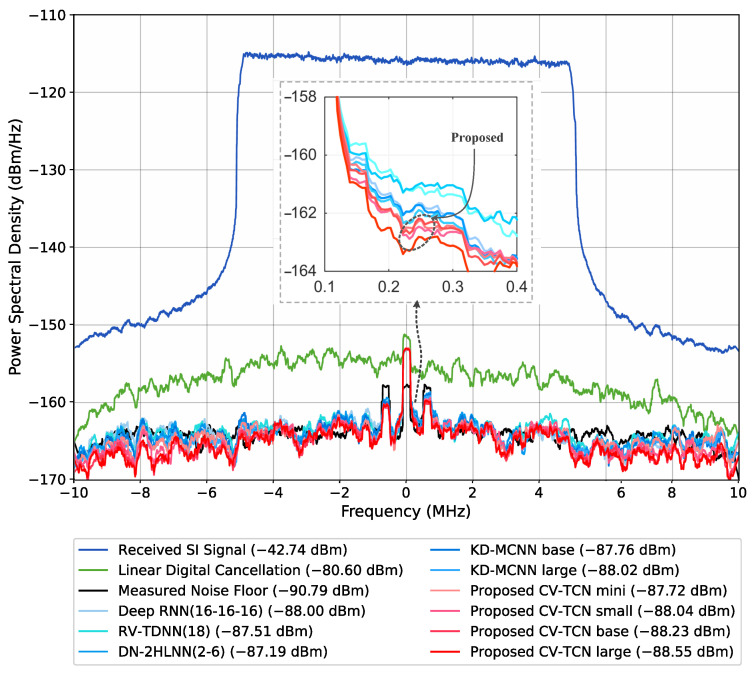
PSD after SIC on Dataset 1.

The power spectral density (PSD) of the residual SI signal after linear and nonlinear cancellations is shown in Figure 5. The results demonstrate that the residual SI signal power is close to the receiver’s noise floor. Particularly with the help of the CV-TCN large canceller, the residual nonlinear SI signal power is decreased from −80.60 dBm to −88.55 dBm, which is only 2.23 dB above the receiver’s noise floor.

### 4.5. Experiment Results for Dataset 2

In this experiment, the CV-TCN models are evaluated on Dataset 2 using Morlet activation function, with jitter augmentation σ = 0.05 and an augmentation factor of 2. A polynomial-based canceller with order *P* = 7 is employed as the baseline. The training configurations for HCRNN, HCRDNN, RNN, RV-TDNN, and CV-TCN are the same as Dataset 1 and the tap number is set to 6 [21].

To evaluate the effectiveness of the Morlet activation function within the CV-TCN model on Dataset 2, five other activation functions are used for ablation studies. All CV-TCN models are trained using the same hyper-parameters as Dataset 1, ensuring a fair comparison. The detailed configurations of the CV-TCN and the corresponding nonlinear SIC performance are presented in Table 4.

As shown in Table 4, the choice of activation function significantly impacts SIC performance. The Morlet consistently achieves superior SIC across all model scales. Notably, Morlet yields a cancellation of 14.54 dB for the mini model, representing an 8.95 dB improvement over ReLU. Unlike Morlet and ReLU-based activations that benefit from increased model capacity, Tanh exhibits performance degradation in big/large models. This deterioration is primarily caused by the vanishing gradient problem.

The results compared to other models are presented in Table 5, where the parameters and inference FLOPs of the proposed and existing NN-based cancellers are the sum of the linear and nonlinear parts [8,9,21]. On Dataset 2, CV-TCN cancellers also achieve superior performance compared to other models under the same inference FLOPs or parameter size.

**Figure 6:**
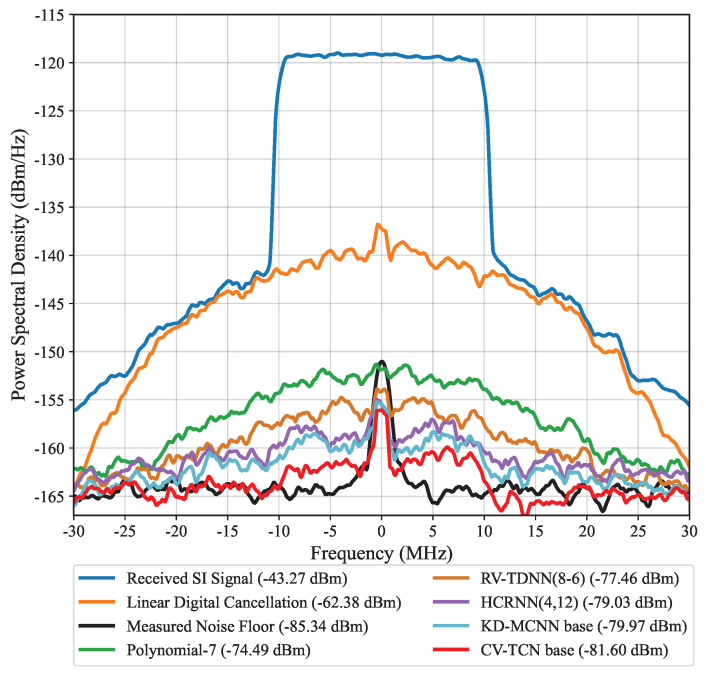
PSD after SIC on Dataset 2.

The PSD of the residual SI signal for Dataset 2 is presented in Figure 6. The figure illustrates that the CV-TCN base canceller can further reduce the residual SI power from −62.38 dBm to −81.60 dBm after linear SIC stage. Notably, the residual SI spectrum after CV-TCN cancellation is close to the noise floor of Dataset 2.

Complex-valued multiplication has higher FLOPs compared to real-valued operations; CV-TCN exhibits a steeper computational scaling curve than real-valued models such as KD-MCNN. Consequently, despite achieving comparable nonlinear cancellation (∼20.35 dB) with fewer parameters, the CV-TCN large canceller needs higher inference FLOPs than the KD-MCNN-large canceller. CV-TCN maintains superior FLOP efficiency only when its parameter count is below approximately 300. Therefore, CV-TCN is preferable for parameter-constrained lightweight SIC scenarios, whereas real-valued alternatives are better suited for FLOP-sensitive applications.

### 4.6. Experiment Results for Dataset 3

In this experiment, the CV-TCN models are evaluated on Dataset 3 with tap number *T* = 13. The configurations are the same as those for Dataset 1 which are shown in Table 2. The linear cancellation is 33.09 dB for Dataset 3.

Table 6 benchmarks the proposed CV-TCN against polynomial-based cancellers and some existing NN-based cancellers on Dataset 3. Using the P=5 polynomial canceller as baseline, all CV-TCN cancellers achieve superior cancellation with lower parameters and FLOPs. Notably, the CV-TCN mini canceller improves nonlinear SIC ratio by 2.06 dB while reducing parameters and FLOPs by over 60%. Furthermore, the CV-TCN base further enhances nonlinear SIC to 12.24 dB with only 57% of the FLOPs of the baseline.

### 4.7. Discussion

#### 4.7.1. Hyperparameter Analysis

As shown in Table 2, SIC performance improves significantly as the number of CV-TCN layers increases from 3 to 5. However, when the depth exceeds five layers, the model suffers from overfitting [23] and yields marginal performance gains.

Similarly, increasing the channel number enhances the model’s capacity to improve cancellation performance. Nevertheless, as shown in Table 4, further widening the convolutional channels introduces a substantial increase in computational complexity, but negligible performance gains.

Thus, the CV-TCN’s depth and width must be carefully selected to balance SIC performance with computational efficiency. Experimental results demonstrate that CV-TCN achieves optimal trade-off when its parameter count remains below approximately 300.

#### 4.7.2. Advantages of CV-TCN

The CV-TCN leverages the TCN architecture to minimize the inference complexity by caching historical computation results to avoid recalculations. By employing complex-valued parameters, the CV-TCN reduces the number of network weights. The introduction of the Morlet wavelet activation function enhances the model’s capability to represent and process the baseband SI signals, achieving a cancellation performance gain of 0.4∼1.4 dB over ReLU activations.

#### 4.7.3. Disadvantages of CV-TCN

The CV-TCN also has some limitations. Implementing the CV-TCN can be more complex than traditional cancellation techniques, requiring a deeper understanding of both the TCN architecture and CVNNs.

Because the CV-TCN requires additional storage for cached historical results and necessitates extra memory read/write bandwidth, the hardware overhead becomes non-negligible on FPGA and DSP platforms operating at high sampling rates (e.g., 100 MHz).

Taking the CV-TCN Base model on Dataset 2 as an example, the intermediate storage is summarized in Table 4. For each clock, the system must read 24 parameters (where each complex value consists of two real-valued parameters) and write 12 intermediate outputs. Assuming a 16-bit (float16) representation, this needs a read bandwidth of 3.84 GB/s (24 × 80 MHz × 2 Bytes) and a write bandwidth of 1.92 GB/s (12 × 80 MHz × 2 Bytes). Such high memory access rates impose a substantial burden on memory interfaces.

Although recent studies have proposed hardware-software co-design optimizations for TCN inference on FPGAs [24,25], these solutions are primarily tailored for general-purpose rather than wireless communication. To bridge this gap, low-bit quantization can be employed to reduce memory bandwidth.

In TCNs, deeper layers benefit more from caching in terms of FLOP reduction. In bandwidth-constrained scenarios, we can prioritize caching the intermediate data of deeper layers where the receptive field is large and recomputation is expensive, and recompute the intermediate data of shallower layers on the fly. This provides a flexible trade-off between computational complexity and memory bandwidth.

## 5. Conclusions

The CV-TCN is proposed in this work to model and cancel nonlinear SI in FD transceivers for ISAC systems. By leveraging complex-valued parameters, temporal convolution, and wavelet activation, the proposed architecture achieves strong nonlinear modeling capacity with low inference complexity. Experimental results demonstrate that the CV-TCN outperforms existing polynomial and NN-based cancellers under equivalent resource constraints. The proposed method reduces residual nonlinear SI by 7.95 dB, bringing the residual SI power down from −80.60 dBm to −88.55 dBm.

In future work, it is advisable to test the performance of the proposed CVNN-based canceller on FD-based ISAC hardware and explore the application of CVNNs to address other challenges in FD systems.

## Figures and Tables

**Table 1:** Summary of the dataset parameters [8,20,21].

Parameter	Dataset 1	Dataset 2	Dataset 3
Modulation	QPSK	QPSK	16QAM
Bandwidth	10 MHz	20 MHz	40 MHz
Carrier number	1024	2048	4096
Sampling frequency	20 MHz	80 MHz	100 MHz
Average transmit power	10 dBm	32 dBm	30 dBm
Passive analog SIC	53 dB	15 dB	50 dB
Active analog SIC	N/A	50 dB	N/A
Total analog SIC	53 dB	65 dB	50 dB
Transmit/receive antennas	1T1R	1T1R	1T1R
Channel	Real	Real	Rician (k-factor = 10)
Dataset size	20,480 samples	20,480 samples	20,480 samples

**Table 2:** Nonlinear SIC with different activation functions for the proposed CV-TCN cancellers.

CV-TCN	$C_{\mathrm{out}},d,K$	Par.	Add. Storage	Inf. FLOPs	ReLU	LeakyReLU	Tanh	Sigmoid	Mish	Morlet
tiny	[2,2,6], [3,1,3], [1,1,1]	78	8	330 + 10$F_{Act}$	4.90	5.12	5.83	3.27	5.86	**5.95**
mini	[2,1,10], [3,1,2], [1,1,3]	94	16	410 + 10$F_{Act}$	5.48	5.45	6.75	3.61	6.05	**6.92**
small	[2,1,10], [4,1,2], [1,1,3]	110	20	480 + 12$F_{Act}$	6.15	6.07	7.02	4.86	6.84	**7.23**
base	[2,1,8], [3,1,3], [3,1,3], [1,1,2]	152	26	670 + 16$F_{Act}$	6.23	6.15	7.12	4.41	7.11	**7.47**
big	[3,1,6], [4,1,3], [4,1,3], [4,1,3], [1,1,2]	348	52	1580 + 30$F_{Act}$	7.00	6.98	7.48	6.94	7.44	**7.56**
large	[6,1,6], [6,1,3], [6,1,3], [6,1,3], [1,1,2]	794	84	3720 + 48$F_{Act}$	7.43	7.49	7.69	7.39	7.72	**7.82**
huge	[6,1,4], [8,1,3], [8,1,3], [8,1,3], [8,1,3], [1,1,2]	1598	136	7600 + 76$F_{Act}$	7.48	7.55	7.68	7.45	7.75	**7.87**

Inference FLOPs: $F_{Act}$ for ReLU, LeakyReLU, Tanh, Sigmoid, Mish and Morlet is 1, 2, 5, 3, 10, and 5, respectively. The bold values indicate the best nonlinear SIC performance achieved by each activation function.

**Table 3:** FLOPs and parameter reduction for CV-TCN and existing NN-based cancellers compared to polynomial-based (*P* = 5) canceller on Dataset 1.

Network	Total Canc. (dB)	Nonlinear Canc. (dB)	Par.	Inf. FLOPs	Par. Reduction	FLOP Reduction
Polynomial (P= 5)	44.45	6.59	312	1558	−	−
**KD-MCNN tiny1**	**44.01**	**6.15**	139	**416**	−55.44%	**−73.30%**
**KD-MCNN tiny2**	**44.19**	**6.33**	145	**436**	−53.53%	**−72.02%**
MWGS(12,5)	44.40	6.54	212	1026	−32.05%	−34.15%
HCRDNN(5,12)	44.41	6.55	223	725	−28.53%	−53.47%
DN-2HLNN(2-6)	44.44	6.58	130	608	−58.33%	−60.98%
HCRDNN(7,11)	44.44	6.58	248	700	−20.51%	−55.07%
LWGS(9)	44.48	6.62	162	782	−48.08%	−49.81%
CV-TDNN(7)	44.50	6.64	238	1166	−23.72%	−25.16%
HCRNN(9)	44.50	6.64	229	745	−26.60%	−52.18%
DN-2HLNN(2-7)	44.50	6.64	138	638	−55.77%	−58.54%
DN-2HLNN(2-4-5)	44.51	6.65	166	782	−46.79%	−49.81%
KD-MCNN mini1	44.57	6.71	157	634	−49.68%	−59.31%
**CV-TCN tiny**	**44.62**	**6.76**	**104**	**510**	**−66.67%**	**−67.27%**
CV-TDNN(4-4-4)	44.63	6.77	228	1106	−26.92%	−29.01%
RV-TDNN(10-10-10)	44.73	6.87	538	1120	+72.44%	−28.11%
KD-MCNN mini2	44.73	6.87	175	686	−43.91%	−55.97%
RV-TDNN(18)	44.76	6.90	550	1156	+76.28%	−25.80%
Polynomial (P= 7)	44.80	6.94	520	2598	+66.67%	+66.75%
KD-MCNN small	44.90	7.04	201	790	−32.37%	−49.29%
RNN(20)	44.94	7.08	528	1210	+69.23%	−22.34%
**CV-TCN mini**	**44.98**	**7.12**	**120**	**590**	**−61.54%**	**−62.13%**
KD-MCNN base	45.02	7.16	241	1000	−22.76%	−35.82%
Deep RNN(16-16-16)	45.27	7.41	1420	3106	+355.13%	+99.36%
KD-MCNN large	45.28	7.42	354	1390	+13.46%	−10.78%
**CV-TCN small**	**45.29**	**7.43**	**136**	**670**	**−56.41%**	**−57.00%**
KD-MCNN huge	45.45	7.59	535	2110	+71.47%	+35.43%
**CV-TCN base**	**45.49**	**7.63**	**178**	**880**	**−42.95%**	**−43.52%**
KD-MCNN teacher	45.56	7.70	996	3318	+219.23%	+112.97%
**CV-TCN big**	**45.65**	**7.79**	**374**	**1860**	**+19.87%**	**+19.38%**
**CV-TCN large**	**45.81**	**7.95**	**820**	**4090**	**+162.82%**	**+162.52%**
**CV-TCN huge**	**45.81**	**7.95**	**1624**	**8110**	**+420.51%**	**+420.54%**

The bold values indicate that the canceller achieved nonlinear cancellation performance with the lowest parameters or inference FLOPs compared to other NN-based cancellers [9,15].

**Table 4:** Nonlinear SIC performance with different activation functions for the proposed CV-TCN cancellers on Dataset 2.

CV-TCN	$C_{\mathrm{out}},d,K$	Par.	Add. Storage	Inf. FLOPs	ReLU	LeakyReLU	Tanh	Sigmoid	Mish	Morlet
mini	[3,1,4], [1,1,3]	50	12	210 + 6$F^{Act}$	5.48	5.59	4.70	4.04	4.95	**14.54**
small	[2,1,3], [4,1,3], [1,1,2]	90	16	380 + 12$F^{Act}$	9.87	12.55	13.27	7.52	12.64	**16.64**
base	[3,1,3], [6,1,3], [1,1,2]	170	24	750 + 18$F^{Act}$	15.45	15.43	16.12	9.71	18.14	**18.43**
big	[4,1,3], [8,1,3], [1,1,2]	274	32	1240 + 24$F^{Act}$	17.96	17.60	15.23	11.65	**19.41**	19.40
large	[5,1,3], [10,1,3], [1,1,2]	402	40	1850 + 30$F^{Act}$	18.90	18.62	14.93	12.01	19.74	**20.11**

The bold values indicate the best nonlinear SIC performance achieved by each activation function.

**Table 5:** FLOPs and parameter reduction for CV-TCN and existing NN-based cancellers compared to polynomial-based (*P* = 7) canceller on Dataset 2.

Network	Total Canc. (dB)	Nonlinear Canc. (dB)	Par.	Inf. FLOPs	Par. Reduction	FLOPs Reduction
Polynomial (P= **7**)	30.75	11.65	240	1198	−	−
HCRNN(4,9)	33.74	14.63	158	405	−34.17%	−66.19%
RV-TDNN(8-6)	34.07	14.96	184	386	−23.33%	−67.78%
**CV-TCN mini**	34.11	**15.00**	**62**	**300**	**−74.17%**	**−74.96%**
HCRDNN(3,7,11)	34.61	15.50	208	506	−13.33%	−57.76%
**KD-MCNN small**	34.88	**15.77**	113	**486**	−52.92%	**−59.43%**
RV-TDNN(7)	35.24	16.13	242	510	+0.83%	−57.43%
CV-TDNN(7)	35.43	16.32	126	564	−47.50%	−52.92%
HCRNN(4,12)	35.67	16.56	236	558	−1.67%	−53.42%
CV-TDNN(4-4-4)	35.78	16.67	158	684	−34.17%	−42.90%
RNN(20)	36.53	17.42	514	1040	+114.16%	−13.19%
CV-TDNN(8-6)	36.65	17.54	246	1108	+2.50%	−7.51%
KD-MCNN base	36.69	17.58	215	780	−10.42%	−34.89%
HCRDNN(4,9,12)	36.77	17.66	284	645	+18.33%	−46.16%
**CV-TCN small**	36.98	**17.87**	**102**	**500**	**−57.50%**	**−58.26%**
**RV-TDNN(12-10-6)**	37.53	**18.42**	372	**760**	+55.00%	**−36.56%**
**CV-TCN base**	38.33	**19.22**	**182**	**900**	**−24.17%**	**−24.87%**
**KD-MCNN big**	39.06	**19.95**	316	**1104**	+31.67%	**−7.85%**
RV-TDNN(20-20-12)	39.11	20.00	964	1920	+301.67%	+60.27%
**CV-TCN big**	39.24	**20.13**	**286**	**1420**	**+19.17%**	**+18.53%**
**KD-MCNN large**	39.44	**20.33**	501	**1610**	+108.75%	**+34.39%**
**CV-TCN large**	39.48	**20.37**	**414**	**2060**	**+72.50%**	**+71.95%**

The bold values indicate that the canceller achieved nonlinear cancellation performance with the lowest parameters or FLOPs compared to other NN-based cancellers. ReLU is used as activation function in HCRNN, HCRDNN, RNN and RV-TDNN [8,9].

**Table 6:** FLOPs and parameter reduction for CV-TCN and existing NN-based cancellers compared to polynomial-based (*P* = 5) canceller on Dataset 3.

Network	Total Canc. (dB)	Nonlinear Canc. (dB)	Par.	Inf. FLOPs	Par. Reduction	FLOP Reduction
Polynomial (P= 5)	39.95	6.86	312	1558	-	-
RV-TDNN(18)	39.59	6.50	550	1156	+76.28%	−25.80%
CV-TDNN(7)	39.73	6.64	238	1166	−23.72%	−25.16%
HCRDNN(7,11)	39.94	6.85	248	700	−20.51%	−55.07%
Polynomial (P= **7**)	40.67	7.58	520	2598	+66.67%	+66.75%
KD-MCNN small	40.71	7.62	201	790	−32.37%	−49.29%
CV-TDNN(4-4-4)	40.73	7.64	238	1166	−23.72%	−25.16%
RNN(20)	40.82	7.73	528	1210	+69.23%	−22.34%
RV-TDNN(10-10-10)	41.16	8.07	538	1120	+72.44%	−28.11%
KD-MCNN base	41.82	8.73	241	1000	−22.76%	−35.82%
**CV-TCN mini**	42.01	**8.92**	**120**	**590**	**−61.54%**	**−62.13%**
Deep RNN(16-16-16)	42.05	8.96	1420	3106	+355.13%	+99.36%
KD-MCNN large	42.50	9.41	354	1390	+13.46%	−10.78%
**CV-TCN small**	42.51	**9.42**	**136**	**670**	**−56.41%**	**−57.00%**
KD-MCNN huge	43.06	9.97	535	2110	+71.47%	+35.43%
KD-MCNN teacher	44.42	11.33	996	3318	+219.23%	+112.97%
**CV-TCN base**	45.31	**12.24**	**178**	**880**	**−42.95%**	**−43.52%**
**CV-TCN big**	46.82	**13.73**	**374**	**1860**	**+19.87%**	**+19.38%**

The bold values indicate that the canceller achieved nonlinear cancellation performance with the lowest parameters or FLOPs compared to other NN-based cancellers.

## Data Availability

The datasets analyzed in this study are publicly available. Dataset 1 can be accessed at https://github.com/abalatsoukas/fdnn (accessed on 1 March 2026), and Dataset 2 is available at https://github.com/abalatsoukas/CSI-full-duplex (accessed on 1 March 2026). The simulation-generated dataset used for additional evaluation is available from the corresponding author upon reasonable request.

## Abbreviations

The following abbreviations are used in this manuscript:

ADC	Analog-to-Digital Converter
CVNN	Complex-Valued Neural Network
CV-TCN	Complex-Valued Temporal Convolutional Network
DAC	Digital-to-Analog Converter
FD	Full-Duplex
FLOP	Floating-point operation
IQ	In-Phase and Quadrature
ISAC	Integrated sensing and communication
LNA	Low-noise amplifier
OFDM	Orthogonal Frequency Division Multiplexing
PA	Power Amplifier
PSD	Power Spectral Density
QPSK	Quadrature Phase Shift Keying
RF	Radio Frequency
SI	Self-Interference
SIC	Self-Interference Cancellation
TCN	Temporal Convolutional Network
